# *Artemisiae argyi* Water Extract Alleviates Obesity-Induced Metabolic Disorder

**DOI:** 10.3390/cimb44120420

**Published:** 2022-12-07

**Authors:** Youngji Han, Hae-Jin Park, Min-Kyeong Hong, Mi-Rae Shin, Seong-Soo Roh, Eun-Young Kwon

**Affiliations:** 1 Department of Food Science and Nutrition, Kyungpook National University, 1370 San-Kyuk Dong Puk-Ku, Daegu 41566, Republic of Korea; 2 Center for Food and Nutritional Genomics Research, Kyungpook National University, 1370 San-Kyuk Dong Puk-Ku, Daegu 41566, Republic of Korea; 3 Center for Beautiful Aging, Kyungpook National University, 1370 San-Kyuk Dong Puk-Ku, Daegu 41566, Republic of Korea; 4 Raydel Research Institute, 76, Dongnae-ro, Dong-gu, Daegu 41061, Republic of Korea; 5 Bio Convergence Testing Center, Daegu Haany University, 1 Haanydaero, Gyeongsan-si 38610, Republic of Korea; 6 Department of Herbology, College of Korean Medicine, Daegu Haany University, 64 Gil, 136, sinsincheondo-ro, Suseong-gu, Daegu 42158, Republic of Korea

**Keywords:** type 2 diabetes mellitus, obesity, herbal medicine, natural product, *Artemisiae argyi*

## Abstract

*Artemisiae argyi* is a well-known traditional herbal medicine used in East Asia. Although the antibacterial and anti-inflammatory effects of *A. argyi* have been reported, its efficacy in improving obesity has not been yet evaluated. In this study, mice were fed a normal diet (AIN-93), a high-fat diet (HFD, 60% of kcal from fat), and an HFD with 0.1% of *A. argyi* water extract for 16 weeks. The body weight and body fat in *A. argyi-*fed mice significantly decreased via upregulation of the mRNA expression of fatty acid oxidation-related genes, with a simultaneous decrease in plasma lipid content and leptin levels. *A. argyi* water extract also ameliorated hepatic steatosis by restricting lipogenesis via lowering the activities of fatty acid synthase and phosphatidic acid phosphatase. Consistently, hepatic histological analysis indicated that *A. argyi* water extract decreased hepatic lipid accumulation in accordance with the hepatic H, E and Oil Red O-stained area. Additionally, *A. argyi* ameliorated the impaired glucose homeostasis by increasing the mRNA expression of AMP-activated kinase and glycolysis-related genes. In conclusion, our results indicate that *A. argyi* can be used to treat obesity-related metabolic conditions.

## 1. Introduction

As interest in improving quality of life has increased, the efficacy and safety of pharmaceuticals have also garnered increasing attention [1]. Developed countries have previously evaluated the economic value of resource plants distributed globally and are now focusing on securing more diverse plant species and systematically developing new functional pharmaceutical materials from them [2]. Natural products are basic resources for novel drug development, and research using natural product extracts is in the spotlight [3].

*Artemisia* is a herbaceous plant belonging to the *Asteraceae* family. It is estimated that 300 of about 400 species of this genus are present in Korea, but only about 40 species have been reported thus far [4]. *Artemisiae argyi—*widely consumed in Korea—is rich in flavonoids, polysaccharides, and volatile oils, such as isocoumarin, coumarin, diterpenlactone, flavonoid, phellandrene, couprol, cadinene, cineol, artemisinin, and euphatrin [5,6]. Previous studies have investigated *A. argyi*’s health-promoting properties, concluding the prevention of oxidative stress, cancer, inflammation, osteoporosis, and immunomodulatory and its neuroprotective activities [5,7,8,9,10,11]. However, studies on the efficacy of *A. argyi* water extract in diet-induced obesity are lacking. Therefore, in this study, we explored the potential of *A. argyi* as a dietary or supplemental source to modulate obesity-associated metabolic disorders in diet-induced obese mice. This study highlights the potential application of *A. argyi* water extract as a modulator of obesity-associated metabolic disregulation in functional foods.

## 2. Materials and Methods

### 2.1. Extract Preparation

Artemisiae argyi was purchased from Bonchowon (Yeongcheon, Korea) and a voucher herbarium specimen (DHU-KM-2020-07) was verified at the College of Korean Medicine in Daegu Haany University. The above-ground part of dried Artemisiae argyi (300 g) was extracted by 10× volume of distilled water (100 °C) for 2 h. After filtering the extract with qualitative filter paper (Hyundai Micro, No. 22, 285 mm), the solvent was evaporated in vacuo to obtain powders (with a yield rate of 13.2%). Powder was stored at −80 °C until experimentation. Table 1 shows the chemical composition of the *A. argyi* extract. The total polyphenol content of *A. argyi* was measured with reference to the method of Rama et al. [12]. Amounts of 100 μL of diluted sample solution, 500 μL of Folin–Ciocalteu’s phenol reagent (diluted to 10×), and 400 μL of 7.5% sodium carbonate were mixed and left in the dark for 30 min, and then absorbance was measured using a multi-function microplate reader (765 nm) (infinite M200 Pro, Tecan, Männedorf, Switzerland). Gallic acid (G7384, Sigma-Aldrich, Saint Louis, MO, USA; ≥97.5% purity) was used to plot a standard calibration curve and calculate the total polyphenol content of the sample. The total flavonoid content of *A. argyi* was measured with reference to the method of Jiao et al. [13]. Amounts of 100 μL of diluted sample solution, 300 μL of methanol, 20 μL of 10% aluminum chloride solution, 20 μL of 1 M potassium acetate solution, and 560 μL of distilled water were mixed and left in the dark for 30 min, and then absorbance was measured using a multi-function microplate reader (415 nm). Quercetin (Q4951, Sigma-Aldrich, Saint Louis, MO, USA; ≥95% purity) was used to plot a standard calibration curve and calculate the total flavonoid content of the sample. The eupatilin (SML1689, Sigma-Aldrich, Saint Louis, MO, USA; ≥98% purity) contents of the A. argyi was measured with reference to the method of Xia et al. [14]. The *A. argyi* water extract was dissolved with 50% methanol to 4000 mg/mL, and filtrated through a 0.2 μm PTFE syringe filter (Advantec DIS-MIC-13HP, Toyo Roshi Kaisha, Ltd., Tokyo, Japan). The LC–MS analysis was performed on an Vanquish Horizon UPLC system connected to an Orbitrap Exploris 120 mass spectrometer (Thermo Fisher scientific, Cleveland, OH, USA).

### 2.2. Experimental Animals and Diet

Thirty male C57BL/6J mice (4-weeks-old) were purchased from Jackson Laboratory (Bar Harbor, ME, USA). The animals were maintained in a temperature- (20–23 °C) and light-controlled (12/12-h light–dark cycle) room and fed a pelletized, commercial, non-purified diet for one week after arrival. The mice were randomly divided into three groups (n = 10 per group) and fed the respective experimental diets for 16 weeks: normal diet control (ND, American Institute of Nutrition [AIN]-93 semi-synthetic diet), high-fat diet control (HFD, 60% of kcal from fat, based on the AIN-93G diet), and 0.1% *A. argyi* water extract (AA; HFD with 0.1% *A. argyi* water extract, *w/w*) (Table 2). The dose of the *A. argyi* water extract was determined by previous studies [15,16,17]. The mice had free access to the experimental diet and water during the experimental period. Their food intake was recorded daily, and body weight was monitored biweekly. All animal procedures were approved by the Ethics Committee for Animal Studies at Kyungpook National University, Daegu, Republic of Korea (approval no. KNU-2020-0090).

### 2.3. Sample Preparation

At the end of the experimental period, all the mice were anesthetized with isoflurane (Baxter, United States) Isoflurane was administered in a mixture with oxygen at a constant flow of 0.5–0.7 L/min. The animals were placed in the induction chamber, a plastic container of 20 cm in diameter and 10 cm in height, connected with a polyurethane polyether tube to let in the anesthetic gas mixture. Induction of the anesthesia was achieved by using 4% vaporized isoflurane (Laboratorios Esteve S.A., Barcelona, Spain) in oxygen.

After sacrificed with a 12-h fast, blood samples were collected from the inferior vena cava into a heparin-coated tube for the measurement of plasma parameters. The blood was centrifuged at 1000× *g* for 15 min at 4 °C, and the plasma was separated. After blood collection, epididymal white adipose tissue (WAT), perirenal WAT, retroperitoneal WAT, mesentery WAT, subcutaneous WAT, and liver were promptly removed, rinsed with physiological saline, and weighed. Among them, epididymal WAT and liver were immediately frozen in liquid nitrogen and stored at −70 °C until the analyses of the enzyme activity and RNA.

### 2.4. Plasma and Hepatic Lipid Profile

Plasma triglyceride (TG), total cholesterol (TC), and high-density lipoprotein cholesterol (HDL-C) concentrations were determined using commercial kits (Asan, Seoul, Republic of Korea). Plasma free fatty acid (FFA) content was measured using an enzymatic kit (Wako Chemicals, Richmond, VA, USA). Plasma apolipoprotein (Apo) A-I and Apo B levels were also determined using an enzymatic kit (Eiken, Tokyo, Japan). Hepatic lipids were extracted according to Folch’s method [18], and the content was determined using the same commercial kits used for the plasma lipid profile analysis.

### 2.5. White Adipose Tissue (WAT) and Hepatic Morphology

WAT and liver samples were fixed in 10% buffered formalin, embedded in paraffin, and semi-serial sections at 4 μm-thickness were cut at 1 mm intervals, stained with hematoxylin and eosin (H and E). Frozen hepatic tissue was semi-serial sectioned at 7 μm-thickness with 1 mm intervals stained with Oil Red O, and observed under an optical microscope (Nikon, Tokyo, Japan) under 200× magnification [19,20]. All morphological quantification was performed based on image J using 10 images per group for analysis [21,22].

### 2.6. Plasma Adipokine Content

Plasma leptin, adiponectin, and resistin levels were measured using a multiplex detection kit (Bio-Rad Laboratories Inc., Hercules, CA, USA). The Luminex 200 LabMAP system (Luminex, Austin, TX, USA) and Bio-Plex Manager software (version 4.1.1; Bio-Rad Laboratories, Inc.) were used for the analysis.

### 2.7. Fasting Blood Glucose Level, Intraperitoneal Glucose Tolerance Test, and Homeostatic Index of Insulin Resistance (HOMA-IR)

Animals were fasted for 12 h before undergoing a fasting blood glucose (FBG) determination test and an intraperitoneal glucose tolerance test (IPGTT). The FBG concentration in the blood from the tail veins was measured using a OneTouch Select Plus ^®^ meter glucose analyzer (LifeScan, Milpitas, CA, USA). The IPGTT was performed at the 11th week. Glucose was intraperitoneally injected at 0.5 g·per·kg of body weight, and blood glucose concentrations were determined at 0, 30, 60, and 120 min. HOMA-IR was calculated using the following formula:

HOMA-IR = (fasting glucose (mmol/L) × fasting insulin (µL·U/mL))/22.5 [23].

### 2.8. mRNA Expression Analysis

mRNA extraction was performed as previously described [24]. Total mRNA was reverse transcribed into cDNA using the QuantiTect Reverse Transcription Kit (Qiagen, Hilden, Germany). mRNA expression was quantified via real-time quantitative PCR using the QuantiTect SYBR Green PCR kit (Qiagen) and the SDS7000 sequence-detection system (Applied Biosystems, Foster City, CA, USA). Sequences of the primers used are listed in Supplementary Table S1.

### 2.9. Enzyme Activities in the Liver and Epididymal WAT

Liver and epididymal WAT samples were prepared and analyzed using the method developed by Hulcher and Oleson [25]. Fatty acid synthase (FAS) activity was analyzed as described by Nepokroeff et al. [26]. The activities of glucose-6-phosphate dehydrogenase (G6PD), malic enzyme (ME), carnitine palmitoyl transferase (CPT), and fatty acid oxidation were measured using previously described methods [27,28,29,30].

### 2.10. Statistical Analysis

Data are presented as the mean ± standard error of the mean (SE). All statistical analyses were performed using SPSS version 23.0 (IBM, Chicago, IL, USA). Significant differences between the ND and HFD groups and the HFD and AA groups were determined using the Student’s *t*-test. Differences were considered significant at *p* < 0.05.

## 3. Results

### 3.1. A. argyi Water Extract Supplementation Reduces Body Weight and Body Fat Mass

**Figure 1:**
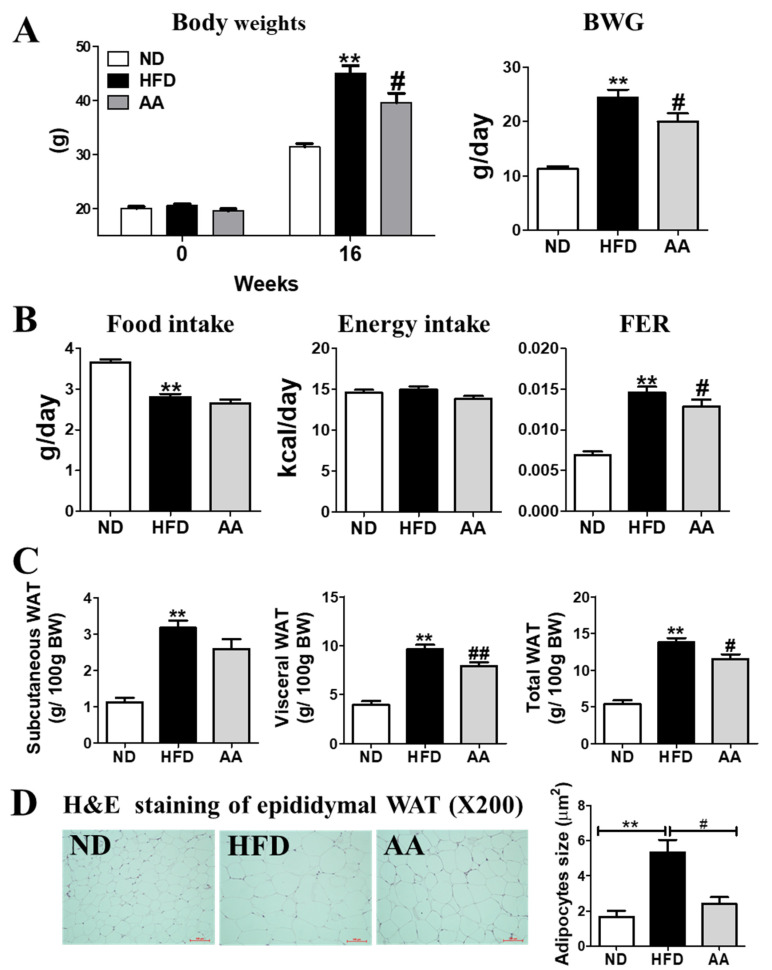
Effect of *Artemisiae argyi* water extract supplementation on diet-induced obesity. (**A**) Body weight and body weight gain; (**B**) food intake, energy intake, and FER; (**C**) adipose tissue weight; and (**D**) adipocyte morphology of the study groups. Data are presented as the mean ± standard error of the mean. Significant differences between HFD and ND are indicated as ** *p* < 0.01. Significant differences between the HFD and AA groups are indicated as # *p* < 0.05, ## *p* < 0.01. ND, normal diet (AIN-93G, n = 10); HFD, high-fat diet (60% kcal from fat, n = 10); AA, HFD + *A. argyi* water extract (1.5%, *w/w*, n= 10); BWG, body weight gain; FER, food efficiency ratio: body weight gain/energy intake per day; WAT, white adipose tissue.

The initial body weights of the mice were not significantly different among the three groups. However, at the end of the experimental period, the HFD-fed mice showed a drastic increase in body weight compared with the ND-fed mice ( 1A). The AA-fed mice had lower body weights than the HFD-fed mice because of the suppression of total body weight gain. No significant differences in food and energy intake between the HFD and AA groups were observed; however, the food efficiency ratio was significantly lower in the AA group than in the HFD group (Figure 1B). Subcutaneous, visceral, and total WAT were significantly increased by HFD consumption (Figure 1C). Consistent with these results, morphological observations revealed that epididymal adipocyte size in the AA group was the smallest of all the three groups (Figure 1D).

### 3.2. A. argyi Water Extract Supplementation Improves Plasma Lipid Profiles and Adipokine Levels by Regulating the Adipocyte mRNA Expression involved in Fatty Acid Oxidation

**Figure 2:**
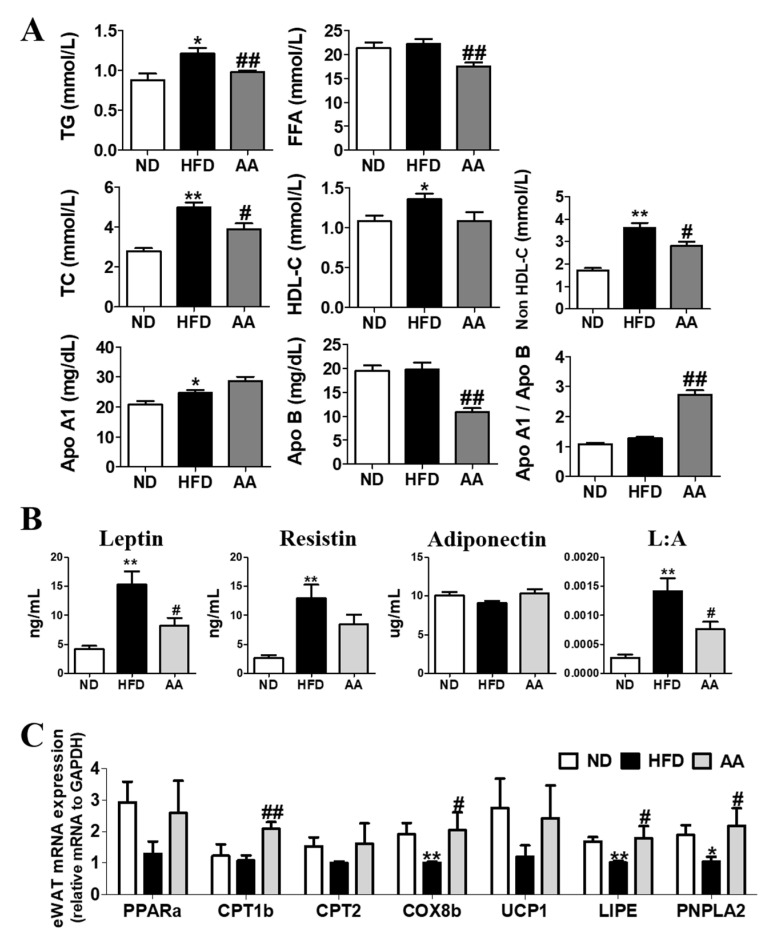
Effect of *Artemisiae argyi* water extract supplementation on diet-induced obesity. (**A**) Plasma lipid profiles, (**B**) plasma adipokine levels, and (**C**) mRNA expression of the epididymal WAT. Data are presented as the mean ± standard error of the mean. Significant differences between HFD and ND are indicated as * *p* < 0.05, ** *p* < 0.01. Significant differences between the HFD and AA groups are indicated as # *p* < 0.05, ## *p* < 0.01. ND, normal diet (AIN-93G, n = 10); HFD, high-fat diet (60% kcal from fat, n = 10); AA, HFD + *A. argyi* water extract (1.5%, *w/w*, n = 10); TG, triglyceride; FFA, free fatty acid; TC, total cholesterol; HDL-C, high density lipoprotein cholesterol; Apo A1, apolipoprotein A-1; Apo B apolipoprotein B; Apo A1/Apo B, Apo A1 and Apo B ratio; L:A, leptin and adiponectin ratio; PPARa, peroxisome proliferator activated receptor alpha; CPT1A, carnitine palmitoyltransferase 1A; CPT2, carnitine palmitoyltransferase 2; COX8b, cytochrome c oxidase subunit 8B; UCP1, uncoupling Protein 1; LIPE, hormone-sensitive lipase; PNPLA2, patatin-like phospholipase domain containing 2.

The lipid profiles of the plasma obtained after a 24-h fast are shown in Figure 2A. Plasma TG, FFA, TC, and non-HDL-C levels were significantly lower in the AA group than in the HFD group. In addition, *A. argyi* water extract supplementation significantly decreased the Apo B level and increased the Apo A1-to-Apo B ratio. The AA group showed significant reductions in visceral and total WAT weights compared with the HFD group. The plasma leptin level and leptin: adiponectin (L:A) ratio were significantly reduced in the AA group (Figure 2B). Moreover, the adipocyte mRNA expression of molecules related to fatty acid oxidation (*CPT1b*, *COX8b*, *LIPE*, and *PNPLA2*) was significantly lower in the AA group than in the HFD group (Figure 2C).

### 3.3. A. argyi Water Extract Supplementation Alleviates Impaired Glucose Metabolism-Related Obesity

**Figure 3:**
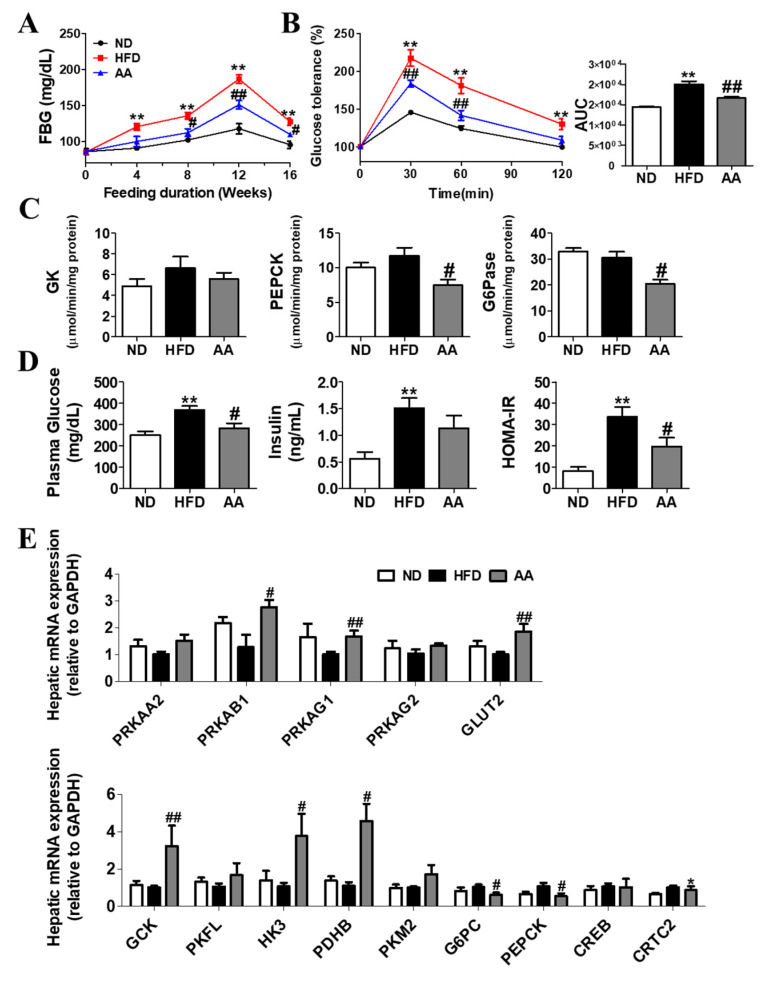
Effect of *Artemisiae argyi* water extract supplementation on diet-induced obesity. (**A**) Fasting blood glucose level; (**B**) intraperitoneal glucose tolerance test and area under the curve; (**C**) activities of hepatic enzymes related to glucose metabolism; (**D**) plasma glucose and insulin levels and homeostatic index of insulin resistance; and (**E**) hepatic mRNA expression of genes related to glucose metabolism. Data are presented as the mean ± standard error of the mean. Significant differences between HFD versus ND are indicated as * *p* < 0.05, ** *p* < 0.01. Significant differences between the HFD and AA groups are indicated as # *p* < 0.05, ## *p* < 0.01. ND, normal diet (AIN-93G, n = 10); HFD, high-fat diet (60% kcal from fat, n = 10); AA, HFD + *A. argyi* water extract (1.5%, *w/w*, n = 10); IPGTT, intraperitoneal glucose tolerance test; FBG, fasting blood glucose; AUC, area under the curve; GK, glucokinase; PEPCK, phosphoenolpyruvate carboxykinase; G6Pase, glucose 6-phosphate; HOMA-IR, homeostatic index of insulin resistance; PRKAA2, 5'-AMP-activated protein kinase subunit alpha-2; PRKAB1, 5'-AMP-activated protein kinase subunit beta-1; PRKAG1, 5'-AMP-activated protein kinase subunit gamma-1; PRKAG2, 5'-AMP-activated protein kinase subunit gamma-2; GLUT2, glucose transporter 2; GCK, glucokinase; PFKL, 6-phosphofructokinase, liver type; HK3, hexokinase 3; PDHB, pyruvate dehydrogenase (lipoamide) beta; PKM2, enzyme pyruvate kinase M2; G6PC, glucose-6-phosphatase, catalytic subunit; GAPDH, glyceraldehyde-3-phosphate dehydrogenase; PEPCK, phosphoenolpyruvate carboxykinase; CREB, cAMP response element-binding protein; CRTC2, CREB regulated transcription coactivator 2.

At the beginning of the 4th week of feeding, FBG levels in the AA group were significantly lower than those in the HFD group (Figure 3A). IPGTT and area under the curve (AUC) results indicated that AA ameliorated glucose intolerance (Figure 3B). Regarding hepatic enzyme activities related to glucose metabolism, *A. argyi* water extract supplementation significantly decreased PEPCK and G6pase activities (Figure 3C). There was no significant difference in plasma insulin levels between the HFD and AA groups; however, plasma glucose levels and HOMA-IR were significantly decreased in the AA group (Figure 3D). The hepatic mRNA expression results are shown in Figure 3E. Regarding the hepatic mRNA expression of AMPK-related genes (*PRKAA2, PRKAB1, PRKAG1, PRKAG2,* and *GLUT2*), *A. argyi* water extract supplementation significantly increased the mRNA expression of *PRKAB1, PRKAG1,* and *GLUT2*. Furthermore, *A. argyi* water extract supplementation significantly upregulated the mRNA expression of genes related to glycolysis (*GCK, HK3,* and *PDHB*) and downregulated the expression of those related to gluconeogenesis (*G6PC* and *PEPCK*).

### 3.4. A. argyi Water Extract Supplementation Alleviates Hepatic Steatosis

**Figure 4:**
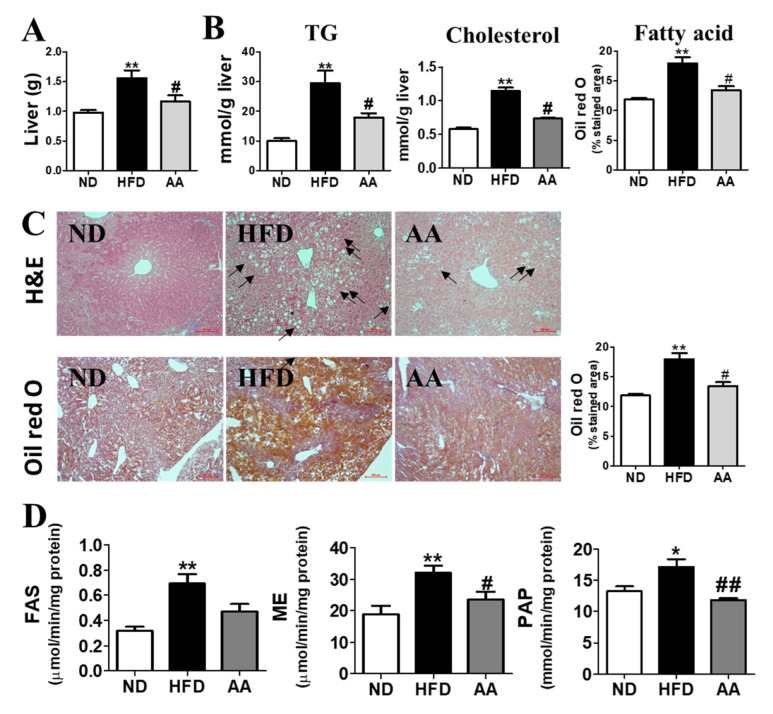
Effect of *Artemisiae argyi* water extract supplementation on diet-induced obesity. (**A**) Liver weight, (**B**) hepatic lipid content, (**C**) hepatic morphology (200× magnification), and (**D**) activities of hepatic enzymes related to fatty acid synthesis. Data are presented as the mean ± standard error of the mean. Significant differences between HFD versus ND are indicated as * *p* < 0.05, ** *p* < 0.01. Significant differences between the HFD and AA groups are indicated as # *p* < 0.05, ## *p* < 0.01. ND, normal diet (AIN-93G, n = 10); HFD, high-fat diet (60% kcal from fat, n = 10); AA, HFD + *A. argyi* water extract (1.5%, *w/w*, n = 10); H and E, hematoxylin and eosin. TG, triglyceride; FAS, fatty acid synthase; ME, malic enzyme; PAP, phosphatidic acid phosphatase.

*Artemisiae argyi* water extract supplementation significantly decreased liver weight and lowered hepatic TG and hepatic cholesterol levels (Figure 4A,B). Hepatic morphological observations and Oil Red O staining revealed reduced lipid formation and accumulation in the AA group compared with that the HFD group (Figure 4C). In particular, the Oil Red O-stained area, which can clearly indicate the presence of lipid droplets, was significantly increased by HFD consumption; however, in the AA group there was a significant decrease in the Oil Red O-stained area compared with the values of the HFD group. Moreover, *A. argyi* water extract supplementation significantly decreased hepatic ME and PAP activities (Figure 4D).

### 3.5. A. argyi Water Extract Supplementation Ameliorates Oxidative Stress

**Figure 5:**
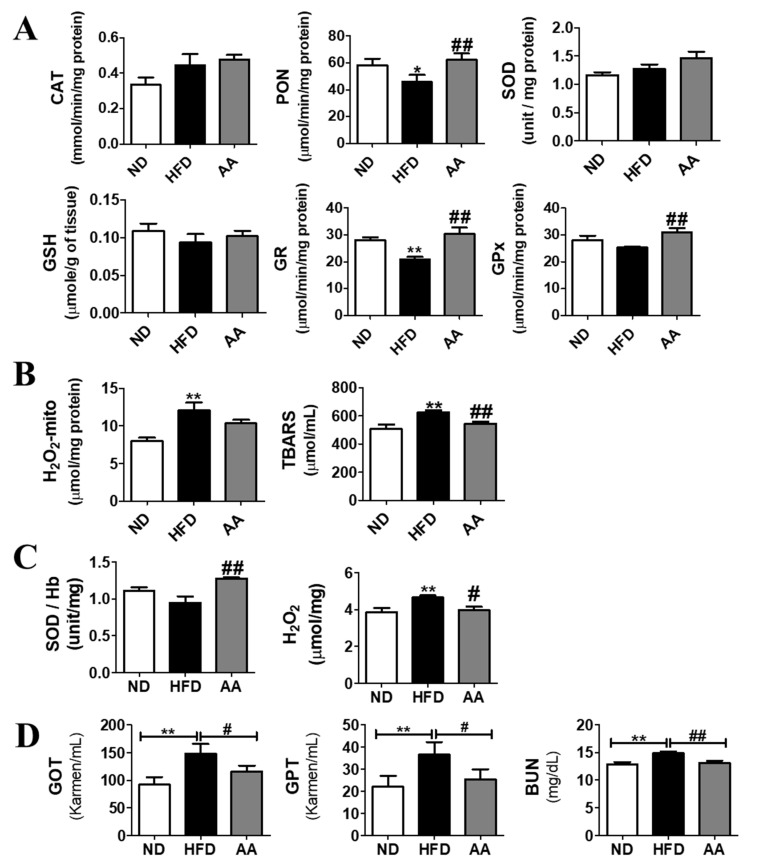
Effect of *Artemisiae argyi* water extract supplementation on diet-induced obesity. (**A**) Activities of hepatic antioxidant enzymes, (**B**) hepatic H_2_O_2_ and TBARS levels, (**C**) erythrocyte SOD and H_2_O_2_ levels and (**D**) plasma liver function index. Data are presented as the mean ± standard error of the mean. Significant differences between HFD versus ND are indicated as * *p* < 0.05, ** *p* < 0.01. Significant differences between the HFD and AA groups are indicated as # *p* < 0.05, ## *p* < 0.01. ND, normal diet (AIN-93G, n = 10); HFD, high-fat diet (60% kcal from fat, n = 10); AA, HFD + *A. argyi* water extract (1.5%, *w/w*, n = 10). CAT, catalase; PON, paraoxonase; SOD, superoxide dismutase; TBARS, thiobarbituric acid reactive substances; GOT, glutamic oxaloacetic transaminase; GPT, glutamic oxaloacetic transaminase; BUN, Blood Urea Nitrogen.

The hepatic antioxidant activity is shown in Figure 5A. Hepatic PON, GR, and GPx activities were significantly higher in the AA group than in the HFD group. In addition, hepatic TBARS levels were significantly lower in the AA group (Figure 5B). Furthermore, SOD activity was significantly increased and endogenous H_2_O_2_ levels in the erythrocytes were significantly decreased in the AA group than in the HFD group (Figure 5C). Plasma concentration of GOT, GPT, and BUN, which are an index of hepatic function, increased in the HFD group compared to the ND group; however, the AA supplement significantly decreased compared to the values of the HFD.

## 4. Discussion

This study investigated the effects of the *A. argyi* water extract on metabolic disorders caused by obesity and its complications. Our findings suggest that *A. argyi* supplementation reduces body weight and body fat without affecting food and energy intake. As shown in Figure 1, the food intake in the AA group was approximately 2.65g, of which the amount of *A. argyi* water extract intake per day was estimated to be 2.65 mg. This animal dose could be translated to a human dose. Based on previous study, the dose of *A. argyi* water extract for adults with a mean body weight of 60 kg was 158 mg, in order to be an effective dose of *A. argyi* water extract. This indicates that the *A. argyi* water extract might reduce body fat through metabolic regulation rather than appetite suppression. Excessive caloric intake and the availability of an energy-dense diet are the main contributors to obesity [31]. HFD has high energy density, thus elevating body weight owing to increased adiposity in various rodent models [32]. We found that body weight, body fat, and adipocyte size were dramatically increased in the HFD group; however, *A. argyi* water extract supplementation significantly reduced these biomarkers in such mice than in those fed only HFD. Furthermore, *A. argyi* water extract significantly increased the mRNA expression of *CPT1b*, *COX8b*, *LIPE*, and *PNPLA2* in the epididymal WAT. Fatty acid oxidation inhibits lipid accumulation by inhibiting the re-esterification of fatty acids to TGs and by using acetyl-CoA from the β-oxidation of fatty acids as respiratory fuel [33]. The anti-adiposity effect of *A. argyi* water extract was supported by the diminished plasma leptin levels and L:A ratio in the present study, as adipokine levels are positively correlated with body fat [34]. Therefore, *A. argyi* water extract can suppress lipid accumulation via activated fatty acid oxidation in the epididymal WAT.

Our findings also suggest that *A. argyi* water extract ameliorates hyperglycemia and insulin resistance caused by HFD-induced obesity, which can be interpreted from two perspectives. First, *A. argyi* water extract ameliorated hepatic steatosis, which could in turn alleviate the impaired blood glucose regulation. Hepatic insulin resistance is a principal component of type 2 diabetes (T2DM) [35]. Impaired hepatic insulin sensitivity induces to increased hepatic gluconeogenesis, hyperinsulinemia, β-cell hypertrophy, and hyperglycemia [36]. Hepatic steatosis is a symptom in patients commonly with T2DM [37] and is closely associated with the long-term consumption of HFD [38]. In the present study, the HFD-fed mice showed significantly increased liver weights and hepatic lipid levels compared with the ND group, whereas they were decreased in the AA group compared with that in the HFD group. Based on previous studies, hepatic lipid accumulation has a positive correlation with liver weights [24,39]. Moreover, Hepatic H and E and Oil Red O staining showed that hepatic lipid accumulation was suppressed in the AA group than in the HFD group. We also observed that the AA group had a significantly decreased hepatic lipid content, as well as hepatic FAS and PAP activities, compared with the HFD group. Thus, *A. argyi* water extract exerts beneficial effects on the initiation and progression of hepatic steatosis. The marked improvements in hepatic steatosis were associated with the decreases in plasma glucose and insulin levels, which is a reflection of ameliorated hepatic insulin sensitivity [40], as evidenced by a reduced AUC for the IPGTT. Additionally, *A. argyi* water extract induced a decrease in hepatic lipid content, along with improved hepatic insulin sensitivity. Moreover, insulin suppressed the gluconeogenesis enzymes activities [41]. Thus, decreased gluconeogenesis and the results in decreased hepatic G6pase and PEPCK activities were suggested to be associated with the improved hepatic insulin sensitivity observed in *A. argyi* water extract-fed, diet-induced obese mice.

In the progression of T2DM, the low-grade inflammation-originated obesity hat is a principal pathophysiological factor related to hyperglycemia and insulin resistance [42,43]. Many studies have reported that various extracts of *A. argyi* possess antioxidant, antibacterial, and immunomodulatory properties [7,44,45]. A previous study suggested that *A. argyi* water extract plays a positive role in lipopolysaccharide-induced oxidative stress by restoring the activities of GPx and SOD, and preventing the increase in nitric oxide concentration caused by the over-activation of total nitric oxide synthase [46]. Consistent with these results, the present study showed that *A. argyi* water extract significantly increased hepatic PON, GR, and GPx activity and decreased hepatic TBARS levels. In addition, hemoglobin SOD activity was elevated, whereas endogenous H_2_O_2_ levels in the erythrocytes were decreased by *A. argyi* water extract supplementation. Furthermore, based on previous studies, a natural product supplement could induce hepatic injury through the interaction with the different cytochrome P-450 isoforms; inflammatory and oxidative activities seem to be the main damage pathway involved in the liver [47,48]. In the present study, *A. argyi* water extract significantly diminished the plasma GOT, GPT and BUN levels. These findings indicate that *A. argyi* water extract ameliorated the oxidative stress caused by obesity by regulating the enzymatic antioxidant system without any hepatic toxicity. In addition, the antioxidant property of *A. argyi* water extract suggests that it can improve glucose homeostasis in diet-induced obese mice.

*A. argyi* is rich in flavonoids, concluding flavones, flavonoids, flavonols, and chalcones [5]. Especially, it has various flavones including jaceosidin, eupatilin, luteolin, apigenin, and neaptin, and so on [49]. Eupatilin is pharmacologically active flavonoid and considered an index component as well as an active compound of *A. argyi*. Many studies supported that eupatilin isolated from *A. argyi* has great antioxidant and anti-inflammation properties in vitro and in vivo and inhibits adipogenesis in 3T3-L1 through the suppression of PPARγ expression [15,50,51,52]. Eupatilin is reported to be contained in A. argyi leaves of 0.46 to 1.22 mg/g [53]. Our experimental material, *A. argyi* water extract, has 0.82 ± 0.01 mg/ g eupatilin. These results suggested that water extraction of *A. argyi* could extract the active component of *A. argyi*. Although further studies are needed to elucidate the exact mode of action of eupatilin in detail, it is clear that *A. argyi* water extract including eupatilin can exert an inhibitory action on body fat accumulation and the initiation of hepatic steatosis or its progression.

## 5. Conclusions

*A. argyi* water extract supplementation has a significant effect on HFD-induced obesity and its complications. The *A. argyi* water extract improved adiposity, suppressed fatty liver caused by diet-induced obesity, and contributed to the recovery of impaired glucose homeostasis and ameliorated inflammation. Therefore, *A. argyi* water extract may be used as a functional food for the prevention of obesity and obesity-related diseases. This compound has no toxic effect in vivo; human trials are expected with the appropriate dosage in the near future.

## Figures and Tables

**Table 1:** Total phenolic, flavonoid, and eupatilin content in the *Artemisiae argyi* water extract.

	Amount
Total polyphenolic content	143.94 ± 1.20 (GAE)/g
Total flavonoid content	23.42 ± 0.01 (QE)/g
Eupatilin content	0.82 ± 0.01 (mg eupatilin/g)

Values represent the mean ± standard error of the mean; GAE, gallic acid equivalents; QE, quercetin equivalents.

**Table 2:** Diet composition for animal experiment.

Ingredient (g)	ND (AIN-93G)	HFD (60 kcal% Fat)	AA (0.1% *A. argyi*)
Casein	200	267	267
Corn starch	397.486	63.381	63.381
Sucrose	100	0	0
Dextrose	132	176	176
Cellulose	50	67	67
Soybean oil	70	33	33
Lard	0	327	327
Mineral mixture ^1^	35	47	47
Vitamin mixture ^2^	10	13	13
TBHQ, antioxidant	0.014	0.019	0.019
L-cystine	3	4	4
Choline bitartrate	2.5	3	3
*Artemisiae argyi*	-	-	1
Total (g)	1000.00	1000.00	1001.00
Calorie (kcal/g)	4000	5332.62	5332.62

^1^ AIN-93G-mineral mixture (Harlan Teklad Co., Madison, WI, USA). ^2^ AIN-93G-vitamin mixture (Harlan Teklad Co., Madison, WI, USA). ND, normal diet (AIN-93G 16 kcal% fat); HFD, high-fat diet (60 kcal% fat); AA (*Artemisiae argyi*), HFD + *A. argyi* water extract 0.1%. TBHQ; tert-butylhydroquinone.

## Data Availability

Not applicable.

## Supplementary Materials

The following supporting information can be downloaded at: https://www.mdpi.com/article/10.3390/cimb44120420/s1, Table S1: Primer list for rt-PCR.
