# Supplementary material for: Artemisiae argyi Water Extract Alleviates Obesity-Induced Metabolic Disorder
Source: Curr Issues Mol Biol. 2022 Dec 7;44(12):6158–71. doi: 10.3390/cimb44120420 (PMC9776687; doi:10.3390/cimb44120420)
Supplement: Supplementary file 1 [file cimb-44-00420-s001.zip › cimb-1984321-supplementary.pdf]

Supplementary Table S1. Primer list for rt-PCR.

Gene	Primer Direction	Primer Sequence (5′- 3′)
COX8b	Forward	TGT GGG GAT CTC AGC CAT AGT
	Reverse	AGT GGG CTA AGA CCC ATC CTG
CPT1b	Forward	TGC CTT TAC ATC GTC TCC AA
	Reverse	AGA CCC CGT AGC CAT CAT C
CPT2	Forward	GCC TGC TGT TGC GTG ACT G
	Reverse	TGG TGG GTA CGA TGC TGT GC
CREB	Forward	GAA GAA GCA GCA CGG AAG AGA
	Reverse	TCT CTT GCT GCC TCC CTG TT
CRTC2	Forward	ATG AAC CCT AAC CCC CAA GAC
	Reverse	CGT TCT CCT CAA TAG CAG GGA
G6PC	Forward	GGA GGA AGG ATG GAG GAA GGA ATG
	Reverse	GGT CAG CAA TCA CAG ACA CAA GG
GAPDH	Forward	TGC AGT GGC AAA GTG GAG AT
	Reverse	TTG AAT TTG CCG TGA GTG GA
GCK	Forward	CAG GAC AGT GGA GCG TGA AGA C
	Reverse	TTA CAG GGA AGG AGA AGG TGA AGC
GLUT2	Forward	GTC AGA AGA CAA GAT CAC CGG A
	Reverse	AGG TGC ATT GAT CAC ACC GA
HK3	Forward	GAG AAC CGT GGA CTG GAC AA
	Reverse	CCA GGA AGG ACA CGT CAC AT
LIPE	Forward	GGC TCA CAG TTA CCA TCT CAC C
	Reverse	GAG TAC CTT GCT GTC CTG TCC
PDHB	Forward	GGA GGG AAT TGA ATG TGA GG
	Reverse	CCA CAG TCA CGA GAT GAT TTG
PEPCK	Forward	TGC CTC TCT CCA CAC CAT TGC
	Reverse	TGC CTT CCA CGA ACT TCC TCA C
PFKL	Forward	CGT TGA GGT AGG AAT ACT TCT GCA
	Reverse	ACC TCT TCC GAA AGG AGT GGA
PKM2	Forward	TGC CGT GAC TCG AAA TCC C
	Reverse	GGC CAA GTT TAC ACG AAG GTC
PNPLA2	Forward	CAA CGC CAC TCA CAT CTA CGG
	Reverse	TCA CCA GGT TGA AGG AGG GAT
PPARα	Forward	GCT GGA GGG TTC GTG GAG TC
	Reverse	CGG TGA GAT ACG CCC AAA TGC
PRKAA2	Forward	CAG AAG ATT CGC AGT TTA GAT GTT GT
	Reverse	ACC TCC AGA CAC ATA TTC CAT TAC C

PRKAB1	Forward	GTT GCT GTT GCT TGT TCC AA
	Reverse	ATA CTG TGC CTG CCT CTG CT
PRKAG1	Forward	TCT CCG CCT TAC CTG TAG TGG A
	Reverse	GCA GGG CTT TTG TCA CAG ACA C
UCP1	Forward	AGA TCT TCT CAG CCG GAG TTT
	Reverse	CTG TAC AGT TTC GGC AAT CCT
